# Cancer-Associated Angiogenesis: The Endothelial Cell as a Checkpoint for Immunological Patrolling

**DOI:** 10.3390/cancers12113380

**Published:** 2020-11-15

**Authors:** Antonio Giovanni Solimando, Simona De Summa, Angelo Vacca, Domenico Ribatti

**Affiliations:** 1 Department of Biomedical Sciences and Human Oncology, Section of Internal Medicine ‘G. Baccelli’, University of Bari Medical School, 70124 Bari, Italy; angelo.vacca@uniba.it; 2 Istituto di Ricovero e Cura a Carattere Scientifico-IRCCS Istituto Tumori “Giovanni Paolo II” of Bari, 70124 Bari, Italy; 3 Molecular Diagnostics and Pharmacogenetics Unit, IRCCS Istituto Tumori Giovanni Paolo II, 70124 Bari, Italy; desumma.simona@gmail.com; 4 Department of Basic Medical Sciences, Neurosciences, and Sensory Organs, University of Bari Medical School, 70124 Bari, Italy

**Keywords:** tumor angiogenesis, endothelium, microenvironment, multiple myeloma, immunotherapy, anti-angiogenesis, adhesion molecules, immune-checkpoint inhibitor

## Abstract

**Simple Summary:**

A clinical decision and study design investigating the level and extent of angiogenesis modulation aimed at vascular normalization without rendering tissues hypoxic is key and represents an unmet medical need. Specifically, determining the active concentration and optimal times of the administration of antiangiogenetic drugs is crucial to inhibit the growth of any microscopic residual tumor after surgical resection and in the pre-malignant and smolder neoplastic state. This review uncovers the pre-clinical translational insights crucial to overcome the caveats faced so far while employing anti-angiogenesis. This literature revision also explores how abnormalities in the tumor endothelium harm the crosstalk with an effective immune cell response, envisioning a novel combination with other anti-cancer drugs and immunomodulatory agents. These insights hold vast potential to both repress tumorigenesis and unleash an effective immune response.

**Abstract:**

Cancer-associated neo vessels’ formation acts as a gatekeeper that orchestrates the entrance and egress of patrolling immune cells within the tumor milieu. This is achieved, in part, via the directed chemokines’ expression and cell adhesion molecules on the endothelial cell surface that attract and retain circulating leukocytes. The crosstalk between adaptive immune cells and the cancer endothelium is thus essential for tumor immune surveillance and the success of immune-based therapies that harness immune cells to kill tumor cells. This review will focus on the biology of the endothelium and will explore the vascular-specific molecular mediators that control the recruitment, retention, and trafficking of immune cells that are essential for effective antitumor immunity. The literature revision will also explore how abnormalities in the tumor endothelium impair crosstalk with adaptive immune cells and how targeting these abnormalities can improve the success of immune-based therapies for different malignancies, with a particular focus on the paradigmatic example represented by multiple myeloma. We also generated and provide two original bio-informatic analyses, in order to sketch the physiopathology underlying the endothelial–neoplastic interactions in an easier manner, feeding into a vicious cycle propagating disease progression and highlighting novel pathways that might be exploited therapeutically.

## 1. Introduction

The interface between malignant cells and neighboring vessels, both recently sprouted during angiogenesis, or resident ones, is one of the pivotal physiological events tangled in the expansion of neoplastic cells and their dissemination [1]. Cancer vessels’ formation is deemed as the result of an angiogenic switch driven by both genetic and epigenetic mechanisms that hijack the tumor trajectory through a full blown self-sustaining entity able to interact with the surrounding niche [2]. The newly formed tumor blood vessels have specific characteristics that allow discrimination from resting blood vessels [3]. They are characterized by rapid proliferation, increased permeability, and disorganized architecture [4]. Initially thought to be a must for the growth and progression of tumors, the formation of new vessels was regarded as one of the hallmarks of both solid [5,6] and hematological malignancies [7,8,9]. However, this has turned out not to be the case, as tumors have been uncovered to also be able to grow without neo-angiogenesis, mainly by co-opting pre-existing vessels, but also through vascular mimicry [10]. Since its discovery by Dr. Judah Folkman, tumor angiogenesis has been proposed as a target for novel tumor therapies [11]. However, the success in the clinic of anti-angiogenic compounds has been limited in contrast to many preclinical positive results obtained in animal models [12]. This is partly determined by heterogeneous vascular and immunological pattern dependencies fueling the boundary between the cancer cells and the endothelium counterpart [13,14].

Solid tumor is made up of a plethora of cell types rather than just a homogeneous mass of cancer cell, such as cancer associated fibroblasts, an heterogenous immune cell infiltrate, and the individual cells that form the blood and lymphatic vessels [15]. The biology of the individual cells that form the tumor vasculature is central to many processes in the tumor microenvironment, providing oxygen and nutrients, forming conduits for metastases, and directly signaling into nearby cancer cells or other stromal cells [16,17]. The niche is also important during the crosstalk with immune cells and the endothelium has been uncovered to be a gatekeeper, representing the first cell type that immune cells contact as they are exiting the circulation into the tumor, but also as they leave the tumor back into circulation [18]. The endothelial cells can thus act as a director in many ways in this process of tumor immune surveillance by its ability to interact directly with immune cells and malignant cells.

The ability to develop an angiogenetic response is a property common to all tissues. Tumor angiogenesis has historically been uncovered to be one of the key hallmarks of cancer [19]. Nonetheless, one of the main problems in comparing the different clinical studies that have used antiangiogenetic therapies is the lack of reliable markers for the assessment of the antiangiogenetic activity and efficacy of the drugs used [20]. Moreover, a tumor response to these drugs, in the form of reduction of tumor mass alone, may not be an appropriate index of the effectiveness of the treatment, owing to the cytostatic nature of the treatment and the potential contribution of the vasculature in promoting tumor immunosuppression [21]. This seems to be related to the chaotic and disorganized nature of the tumor vasculature, but also to a plethora of ancillary mechanisms [22]. Furthermore, the ability of an antiangiogenetic drug to induce a prolonged stabilization of the disease and an increase in survival should be considered more significant in the assessment of the response to antiangiogenetic therapies [23]. Here, we recapitulate the available data from a translational standpoint and support the picture we draw of the pathophysiological dysregulated endothelial–neoplastic interactions with two bio-informatic interrogations that show, on the one hand, a vicious cycle of disease progression and, on the other hand, pinpoint pathways of potential therapeutic interest.

## 2. Antitumor Immunity Impairment: Role of Structural and Functional Abnormalities

Despite its essential role, tumor vasculature is structurally and functionally aberrant, with intercellular junctions and extracellular matrix attachments may not form normally in tumors, leading to impaired monolayer formation and barrier function. Completely chaotic loss of tight junctions between adjacent individual cells in the overlapping endothelium, with odd sprouts being cast across the lumen of tumour vasculature, would be an impediment to proper tumor immune surveillance. These abnormalities also occur at the levels of the vasculature in the individual cells directly interacting with the extracellular matrix (ECM) and the pericytes that typically wrap around the outside of the vessel, providing support and stability; nonetheless, in the tumor microenvironment, pericytes are sparse and they are loosely attached to the surface of the tumour vessels, directly contributing to some of the vascular dysfunction [24]. Consistent with preclinical models, patient tumour vessels are disorganized and half of the vessels do not seem to support blood flow at all; alternatively, blood could be detected pooling and flowing in the opposite direction and the vessel diameters have been uncovered to be atypical; a lower wall shear stress can influence the delivery of drugs and immunotherapy along with impaired cancer immune surveillance due to disorganization in the tumor vessels [25]. Thus, there are many aspects of the cancer niche that make it inhospitable to infiltrating immune cells, thus inspiring several strategies aimed to target different aspects of the tumor microenvironment with the goal of improving both the quantity and the quality of infiltrating immune cells [21]. Defining tumors based on the quantity and the quality of immune cell infiltrates allowed to dissect cancer milieu with abundant immune cells, namely inflamed and cold malignancies, as well as immune cells able to enter the tumor microenvironment despite being suppressed [26]. The cancer endothelium can thus be considered a gatekeeper for leukocyte entry and egress from solid and hematological cancers, triggering a cascade that implicates the leukocyte capture by the vessel wall as well as their rolling along the activated surface, and eventually immune cells arrest; next, in order to spread, the patrolling leukocytes ultimately pass through the endothelial boundary via paracellular routes between two adjacent endothelial cells, also being prone to infiltrate via transcellular route, directly through the endothelial cells cytoplasm [27]. Chemokines and integrins play a pivotal role in extracellular matrix (ECM) degradation. In more detail, integrins as heterodimeric molecules constituted by alpha and beta subunits on the cell surface bind to microenvironmental structures via fibronectin and laminin, while activating degradation pathways such as matrix-metallo-proteinases (MMPs) and urokinase-type plasminogen activator (uPA) [28,29]. Moreover, cell-to-cell and cell–ECM interactions are also mediated by adhesion molecules in both solid [30] and hematological malignancies [31,32]. Modifications in adhesion molecules have been related to invasiveness [33,34], angiogenesis [35,36], and druggable targeting [37,38,39]. Furthermore, the tumor vasculature restricts the infiltration of adaptive immune cells [40,41]. Thus, modifying the tumor vasculature can result in improved immune therapeutic outcome [42]. Consequently, a modern technique such as single cell RNA sequencing has been used to identify diverse subpopulations of tumor-associated endothelial cells [43]. It is conceivable to envision gene expression patterns and individual cells found throughout solid and hematological malignancies and a high grade of modulation in genes implicated in homing, trafficking, and retention of anti-tumor immune cells, corroborating at single-cell level that tumor cells are actively suppressing those pathways important for anti-tumor immunity [43,44].

## 3. Improving Immune–Vascular Crosstalk for Cancer Immunotherapy

The cancer immunotherapy has revolutionized the way we treat neoplastic patients in the last years. Since the first Food and Drug Administration (FDA) and European Medicines Agency (EMA) approval of the immune checkpoint inhibitor (ICI) ipilimumab for melanoma, which targets the anti CTLA-4 checkpoint, an explosion of approval of different ICIs that target a PD1 or programmed death-ligand 1 (PDL1) for a wide range of cancer indications has been observed [45,46]. The ICIs have provided significant clinical benefit including improvement in overall survival for some of the most aggressive and often lethal cancers [47]; however, despite the promising results, the overall objective response rate gained by ICIs as a monotherapy remains suboptimal, ranging between 20 to 30%, and overall survival and toxicity profile still need to be improved [48,49,50]. One strategy applied to accomplish higher clinical response is to generate more effective antitumor shrinkage by combining multiple checkpoints [51]. Nonetheless, the toxicity profile is higher [49,52]. Therefore, there is a growing interest aimed to identify alternative strategies to improve the clinical outcome and antitumor response of ICIs, without significantly increasing the risk of toxicities. In the frame of this thinking, cancer immunotherapy points towards a multifaceted profiling and, given the basic pathophysiology underlying cancer immune surveillance evasion modalities, multiple strategies, besides ICIs-based ones, are aimed at targeting immunosuppressant metabolites [53]. The T cells can shape tumor blood vessels and cancer endothelium and prevent the recruitment and infiltration of the effector immune cells while remodeling the ECM, further inhibiting the migration and infiltration of functional patrolling immune cells [54]. Tumor vasculature actively contributes to the immune suppression, as tumor vessels are highly abnormal and functionally impaired, determining a significant degree of hypoxia, acidosis, and necrosis within the tumor [55]. These pathophysiological mechanisms can lead to the production of immunosuppressive molecules such as small ions, lactate, and reactive oxygen species, all of which work to suppress effective T cytotoxic cell function; at the same time, the production of chemokines and cytokines fosters the differentiation and the activation of immunosuppressive cells such as myeloid derived stem cells (MDSCs) and M2, like tumor macrophages, that also act to inhibit the activities of cytotoxic T cells [56]. Conversely, on the vessel, these mechanisms also downregulate multiple adhesion molecules that are essential for the rolling, adhesion, and transmigration of T cells to enter the cancer milieu [57,58,59], creating a highly immunosuppressive microenvironment, dominated by immune suppressive signals and largely devoid effector T cells. Contrariwise, normalizing tumor vasculature improves T cell infiltration, boosting the immune reaction and halting the immune suppressing environment to a more immune activating phenotype and working in synergy with the cancer immunotherapy.

Anti-vascular endothelial growth factor receptor (anti-VEGFR) pioneered the attempts to normalize tumor vasculature and restore its function, as indicated by tissue perfusion and decreasing intratumoral hypoxia, and fostered further investigations aimed at shaping the intratumoral immune cell phenotype in parallel with vascular normalization [23], while polarizing macrophages throughout and M1 gene-expression phenotype, paralleling an increase in adaptive immune cells’ infiltration in the setting of this antiangiogenic treatment [23,60]. Vascular endothelial growth factor (VEGF) and inflammatory molecules are not merely key proangiogenic elements, but are also immune modulators, which boost vascular formation and cooperate in creating permissive environment in most lethal malignancies, and lead to poor drug response [61,62,63] and survival [19,64]. Remarkably, evidence obtained from pre-clinical and clinical breast cancer models points toward a link between favorable prognostic-related angiogenesis genes and T cell signaling, effective immune cell infiltration that is also pericyte-dependent [65]. In more detail, pericytes seem to be crucial for recruiting immune cells into the tumor niche and orchestrating an immune–vascular crosstalk involving CD4/CD8 T cells and pericytes. Furthermore, to efficiently unleash immune effector cells, Tian et al. uncovered tumor vascular normalization synergism and ICIs (either anti-PD1 or anti-CTLA4 antibodies) to be operative and parallel CD4 T cell activation [65]. Collectively, the interplay between T cells and tumor vasculature primes a CD4 T cell activation and the interferon gamma (IFNγ) production, associated with the normalization of tumor vessels and consequent hypoxia attenuation, reduced intra tumor immunoparesis and further recruitment of bystanders’ immune infiltrates, leading to an even enhanced angiogenesis homeostasis. Contrariwise, pericytes or CD4 T cells elimination and major histocompatibility complex (MHC)II inactivation boosted cancer hypoxia, immunosuppression, and metastatic potential [54,65]. Compelling additional evidence corroborated the existence of close interactions between the tumor endothelium and immune effectors cells with therapeutic implications for ICIs treatment in a colorectal cancer model in an interferon gamma (IFNγ)-dependent fashion [66]. In the frame of this thinking, Zheng et al. highlight the importance of IFNγ receptor signaling in host cell populations for both immune response and vascular tumor homeostasis. Thus, a boosting feedback loop of immune reprogramming and tumor vascular regularization shapes the immunoparetic cancer, frequently rich in immunosuppressive cells and dysfunctional effector T lymphocytes being potentially druggable by ICIs, which can in turn stimulate the regularization of blood vessels and ultimately facilitate the infiltration of effector T cells and improve their function, further halting the immune permissive cancer niche [56].

## 4. Multiple Myeloma (MM) as a Paradigm for Endothelial Gatekeeper Function within the Neoplastic Niche: In Silico Functional Enrichment Study Identifies Prognostic Relevant Gene Profiles in MM Bone Marrow Derived Endothelial Cells

Numerous cell types can be mobilized from the bone marrow and directed to the sites of new vessel formation, where they strengthen the proangiogenic effects [1]. Among them, there are non-hematopoietic bone marrow populations, CD45-, called endothelial progenitor cells (EPCs) [67]. Unlike perivascular cells, which function with paracrine mechanisms by secreting VEGF, endothelial progenitors are incorporated into the wall of nascent vessels, where they differentiate into mature endothelial cells. Being VEGFR-1 positive, they bind VEGF and other proangiogenic factors produced by cancer cells [68]. EPCs facilitate vasculogenesis and are deemed a novel target, particularly at the pre-malignant phase of neoplastic process and in the smoldering stage of disease, fostering the “angiogenic switch”. Moreover, during neoplastic dissemination, EPCs stimulate the shift from subclinical to macroscopic secondary lesions [69]. Hematological cancers represent a paradigmatic condition in which EPCs-mediated priming of cancer angiogenesis takes place, given the close cross talk with the neoplastic clone, and the putative shared ontogeny. Thus, the description of neoplastic-infiltrating EPCs in hematological malignancies may shed more light on a more precise anti-angiogenic strategy, with the advantage of tipping the balance of critical phases of disease progression [70]. Multiple myeloma represents a poster child condition in this regard, being characterized by a multistep natural history, as well as by variable pre-neoplastic stages preceding full-blown disease [70,71].

Multiple myeloma (MM) is a clonal proliferation of malignant plasma cells (PCs) accumulating and disseminating in the bone marrow (BM) with ensuing induction of focal skeletal lesions and osteoporosis driving myeloma bone disease, anemia, renal insufficiency, hypercalcemia [72], higher infection rates [73,74,75], and secondary life-threatening complications [76,77,78]. MM represents an ideal model of colonization and interaction of tumor cells in the bone microenvironment [79,80,81], where the immune-milieu [82,83] and aberrant angiogenesis shape a permissive ecosystem, supporting disease progression via a plethora of autocrine [84,85] and paracrine loops [86,87].

Recently, we demonstrated that bone marrow endothelial cells from both newly diagnosed (NDMM) and relapsed-refractory multiple myeloma (RRMM) patients feed into a vicious cycle orchestrated by aberrant adhesion molecules on the bone marrow endothelial cells and plasma cell surface and correlate with poor clinical prognosis [31,35,88]. Based on this evidence and several pieces of data [89,90], increased adhesion molecules levels have been uncovered to contribute to more aggressive phenotype [29,91]. Direct contact of endothelial cells and endothelial progenitors with MM plasma cells would enhance adhesion molecules levels [92,93]. In silico analysis has been performed on dataset GSE28331 (https://www.ncbi.nlm.nih.gov/geo/query/acc.cgi?acc=GSE28331) [93]. Raw data were RMA normalized, using “affy” package (1.56.0) [94]. The method limma [95] was used to detect differentially expressed genes. The results were considered as statistically significant when adjusted p-value < 0.05. K-means and hierarchical clustering were executed using “Factoextra” (1.0.5) [96], “dendextend” (1.9.0) [97], “colorspace” (1.3-2) [98], and “ggplot2” (2.2.1) [99].

To characterize the adhesion molecules-related angiogenic switch in more detail and to corroborate available at gene-expression level in a broader spectrum of disease phenotype, we interrogated different independent public datasets. Given that mobilization of endothelial precursors cells (EPCs) occurs at the early stages of MM progression [70], preceding MM progression, we selected the GSE28331 data collection.

**Figure 1:**
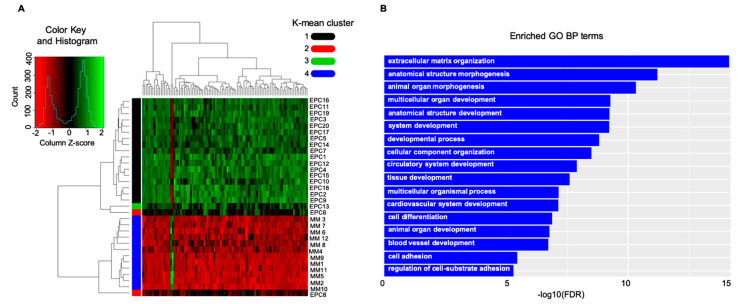
In silico data interrogation points towards a significant crosstalk between the neoplastic-cells and the vasculature counterpart: adhesion-system boosts multiple myeloma (MM)-related angiogenesis in the bone marrow microenvironment. (**A**) Heatmap, showing expression value of the top 100 deregulated genes, includes a dendrogram with two major branches; one containing MM-cells and one EPC sample, and the other grouping the leftover EPCs. (**B**) GO functional enrichment results showed that genes are involved in several biological processes. Cell adhesion and angiogenesis were significantly enriched in the gene network analysis. EPCs: endothelial precursor; GO: gene ontology; BP: biological process.

**Figure 2:**
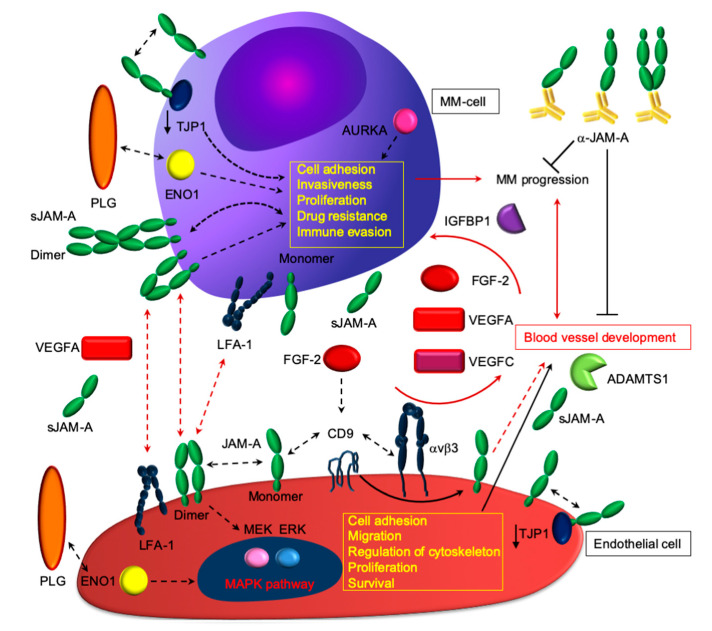
Proposed paradigmatic model of how junctional adhesion molecule-A (JAM-A) plays a pivotal role in angiogenesis, disease progression, and aggressive phenotype. As proof of concept, JAM-A localizes at endothelial tight junctions, in association with the alphaVβ3 integrin. Besides being expressed by MM-cells, JAM-A orchestrates MM angiogenesis: upon stimulation with fibroblast growth factor-2 (FGF-2), the JAM-A-alphaVβ3 complex can dissociate and localizes diffusely along the cell membrane, where it can drive signaling processes, leading to the activation of extracellular signal-regulated MAPK, which leads to angiogenesis and cytoskeleton rearrangement. Trans- homo/heterophilic JAM-A interactions: angiogenesis appears prevalent in MM, as indicated by the results presented in Figure 1 and in [31,35,101]. JAM-A binds heterotypically with lymphocyte function-associated antigen-1 (LFA-1), thus promoting potential interactions of MM-cells and endothelial cells with immune cells. These intricate interactions between ligands and receptors within the MM milieu appear to enhance a pro-survival and immunosuppressive environment, where angiogenesis, immune response, and intrinsic tumor cell resistance depend on each other. ADAMTS: A disintegrin and metalloproteinase with thrombospondin motifs 1; AURKA: Aurora kinase A; CD9: CD9 molecule; ENO1: Enolase 1; FGF-2: fibroblast growth factor-2; LFA-1: lymphocyte function-associated antigen 1; MAPK: mitogen-activated protein kinase; PLG: plasminogen; TJP1: tight junction protein-1; αVβ3: integrin alpha V beta 3; VEGFA: vascular endothelial growth factor A. See the results and [35] for additional details.

Next, determining whether MM EPCs could be distinguished from MM-cells according to the natural grouping of their gene expression profiles, we analyzed publically available data from 20 EPC and 12 MM-cell samples (GSE28331). The analyses clearly split the MM-cells and EPCs into two branches (heatmap, Figure 1A), according to the expression values of the top 100 different regulated genes. Over-expression of angiogenic genes in EPCs deemed statistically significant and relevant for pro-angiogenic biological processes increased expression of angiogenic genes in EPCs deemed statistically significant and relevant for pro-angiogenic biological processes (Figure 1A,B). Based on these different expression patterns, we performed an enrichment pathway and functional annotation analysis (Figure 1B). These in silico unpublished data together with the previously described autocrine loop pinpoint that the cell adhesion molecules have noteworthy qualities; they can be involved in the homophilic network on two opposing cell types; moreover, adhesion molecules are shed as soluble isoforms being able to bind to cell-bound isoforms, which in turn even enhances its binding capacity (Figure 2). What develops is a vicious cycle of neoplastic MM cells expressing and shedding adhesion molecules, increasing membrane-bound expression on the endothelium and boosting angiogenesis. In turn, increasing numbers of activated vessels can increasingly bind cancer cells, which promptly catch enhanced space within the neoplastic milieu for contact-mediated interactions [35,100] (Figure 2).

**Figure 3:**
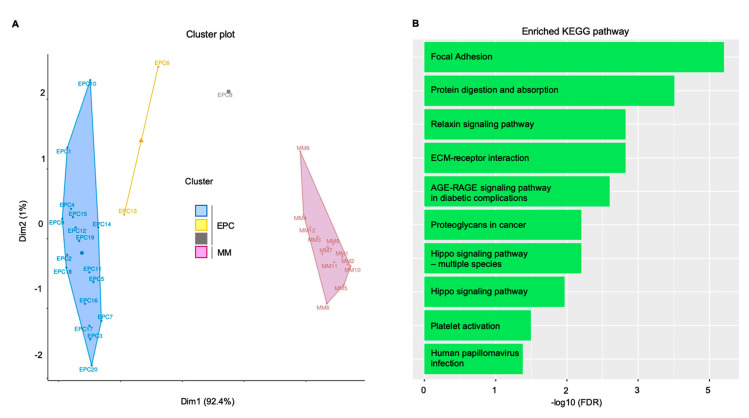
In silico validation confirmed the pivotal role of focal adhesion in sustaining the MM clone. Endothelial cells and MM gene expression supported the bioinformatic findings. (**A**) K-mean clustering results represented as distribution of samples in clusters. (**B**) Gene network functional enrichment: histogram representation of significantly enriched KEGG pathways. Overall, focal adhesion and extracellular matrix (ECM)–receptor interaction confirmed the in vitro and ex vivo evidences. Dim: dimension. KEGG: Kyoto Encyclopedia of Genes and Genomes; AGE: advanced glycation end products; EPC: endothelial progenitor cells; FDR: false discovery rate; MM: multiple myeloma plasma cells; RAGE: receptor for advanced glycation endproducts.

The K-means clustering from the above-mentioned GSE28331 dataset (Figure 3A) showed highly ranked enriched biological processes including blood vessel formation, cell adhesion, and developmental processes; the network analysis highlighted a significant enrichment for focal adhesion and matrix-receptor interaction Kyoto Encyclopedia of Genes and Genomes (KEGG) pathways (Figure 3B).

Consequently, using several pre-clinical models [30,88,102], blocking the adhesion system seems to halt blood vessel formation, reduce adhesion-mediated networks, and weaken neoplastic disease progression. These therapeutic effects of interfering with the adhesion system were observed in translational animal models, not in patients and, therefore, must be interpreted with caution. Nevertheless, these pieces of evidence may be a warning of a pivotal druggable targets of MM and, more generally, microenvironment addicted malignancies that might be investigated therapeutically.

The dysregulated endothelial–neoplastic interactions sketched by our bio-informatic investigations show, on the one hand, a vicious cycle of disease progression and, on the other hand, point out pathways of potential therapeutic interest. These gene expression profiles were observed in one model of disease, and thus must be interpreted with caution and need further validation on a broad spectrum of malignancies. Nonetheless, solid and hematological malignancies share common mechanisms involving the cross talk between the cancer endothelium and the immune microenvironment, as summarized in Table 1.

## 5. Measuring T Cell Exit from Tumors: How Do Lymphatic Vessels Shape the Intratumoral Repertoire 

The lymphatic vasculature is a hierarchical network of vessels found within nearly all peripheral tissues. The main function of lymphatic vessels is to unidirectionally transport interstitial fluids proteins and leukocytes from tissue periphery to the draining lymph nodes structure [139]. The organization of lymphatic vessels is uniquely designed to carry out transport functions in tissues and allow leukocyte egress in order to target solid and hematological malignancies [58,140]. The lymphatic system has been explored by several methods, such as in vivo approaches aiming to quantify leukocyte egress upon the uptake of a labeled tracer and microparticle injection [141]. Specifically, pre-labeled cells are injected into the skin, as the most convenient site, and then labeled cells are detected in the draining lymph node, while quantifying the number of migrating lymphocytes as a readout for the amount of egress occurring [141]. Alternatively, interstitial adoptive transfer and intravital microscopy served as lymphatic vasculature investigating tools [142]. In more detail, intravital microscopy allows to actually visualize and track the movements of pre-labeled leukocytes within tissues as well perceive them entering into pre-labeled lymphatics [142]. Moreover, photoconvertible mice using cell type-specific expression of photoconvertible fluorescent protein Kik Green-Red offered a novel strategy to T cell egress quantification in vivo [143,144]. Remarkably, tumor egressed immune cells are transcriptionally distinct from intratumoral T cells [145] and the CD8 T cells seem not to express markers of exhaustion [146]. Of note, a physical barrier to egress enhances adoptive T cell therapy efficacy in preclinical models [130]. On top on this, T cell egress from tumors can represent a potential mechanism of immune escape. Nonetheless, the limitations to all these methods are represented by biases in selecting cell types prone to be evaluated for the egress, while the application of a tracer only allows to track cells that can uptake that tracer, namely phagocytic cells. Conversely, by adoptive transfer models, the major caveat is the labeling, limiting the assay to two or three cell types at a time [147]. Even using intravital microscopy, a limited number of labeled leukocytes can be tracked at a single time, and it can also be time-consuming and low throughput for tracking immune cell egress in vivo [147]. Thus, by elucidating the mechanisms that govern egress, it is not only possible to gain a significantly better understanding of how the immune landscape of a tumor is formed, but also to manipulate egress mechanisms in a therapeutically beneficial way [148]. Nonetheless, the translational value of the available finding is still debated and standard histological analysis or flow cytometry profiling of intratumoral leukocyte pools does not really provide any information regarding leukocyte trafficking dynamics [149].

To overcome these caveats, promising new avenues have recently been optimized to study the fate of tumor infiltrating immune cell populations, cancer metastasis, migration patterns of alloreactive T cells, or the dynamics and plasticity of immune cell subsets in different scenarios such as infection, inflammation, and immunotolerance using the in vivo photoconvertible fluorescence protein “kaede” transgenic mice [143]. The unique property of kaede protein is that it is influenced by violet light pulse exposition. This state-of-the-art method uncovered lymph nodes to be heavily infiltrated with myeloid cells, predominantly inflammatory monocytes and macrophages [150]. However, some lymphocytes in these tumors are also present and the egressing population seems mostly represented by CD4 and CD8 T cells [150]. Collectively, the available shreds of evidence point toward a vicious cycle between the lymph nodal endothelium and the patrolling immune cells, implying that egressed and retained T cells differ substantially. An acquisition of markers associated with T cell exhaustion in cells that are retained within the tumor, indicated by high expression of PD1 Tim3 and CD 39, characterizes lymphocytes that are also unable to produce effector cytokines such as interferon gamma and TNF alpha. Contrariwise, T cells that have passed through the tumor and exited to the draining lymph nodes are not expressing markers of exhaustion and retain their ability to produce effector cytokines [151]. It might be advantageous to keep these tumor-specific T cells within the tumor for a much longer period of time, potentially improving their function. Despite that direct translation of subclasses based on the vascular phenotype into clinical decision-making is yet to be achieved, these findings may also point towards a potential Achilles’ heel of multiple cancer that might be exploited therapeutically.

## 6. Boosting Cancer Immunotherapy Using Anti-Angiogenics: Therapeutic Windows and Challenges Offered by the Visualization and Reprogramming of the Tumor Milieu

Across the timeline of the development of various imaging techniques, both clinical and preclinical models greatly contributed to the imaging of tumor vasculature and microenvironment [152,153,154,155,156]. The translational value of imaging tumor blood vessels allowed to identify the abnormal microvasculature, visualizing the shape and diameter, the vessel wall, the abnormal branching, and even the blood flow, characterizing the level of heterogeneity in vivo [157]. Based on these pieces of evidence, cancer vasculature appears to be functionally abnormal [158], corroborating previous findings regarding abnormal blood flow as a consequence of aberrant vessel formation [158,159]. While comparing with normal vessels in the cancer tissue, there is a lack of correlation between the size of the vessels’ diameter and red blood cells velocity [158,159]. Remarkably, the next generation of experimental immunodiagnostics in cancer model also provided imaging understandings regarding immune cell trafficking in tumour vessels, namely monocytes, interacting with the vessel wall [155] and leading to patrolling immune cells’ recruitment. From the above mentioned standpoint, the traditional anti-angiogenesis can deeply affect anti-tumour immunity, as full doses of drugs shrink the tumor, leading to cancer hypoxia and priming immune suppressive cells’ infiltration [160]. A wise use of therapeutic strategies halting the cancer angiogenesis must thus take into account the abnormal metabolic microenvironment characterizing a heterogeneous oxygenation [161,162]. Assessing oxygenation in the different layers of tumour pinpoint that there is a progressive increase of nutrient and oxygen levels across the inner depth [162], thus fueling genomic instability [163], the cancer progression (PD) [16], the switch to anaerobic metabolism [164], as well as the epithelial–mesenchymal transition, metastases [165], and the induction of cancer “stem cell” phenotype [166]. Hypoxia is a hallmark of cancer, inducing many abnormalities with prognostic consequences linked to defects in apoptosis and autophagy [167,168] and the resistance to radio-chemotherapy [169,170,171] and immunotherapy [13,172,173] likewise hamper the cancer aggressive phenotype acquisition, while shaping a pro-angiogenic, inflamed, and immunosuppressive neoplastic ecosystem [154,174,175]. Consequently, it is necessary either to target many different actors on the scene within the neoplastic niche or attempt to homogenize the cancer heterogeneity [161].

### Hypoxia as a Key Factor for Angiogenesis and Immune Equilibrium

Sufficient oxygen pressure is required for our organs to function properly. Conversely, insufficient oxygen supply is a prominent feature in various pathological processes, including tumor development and metastasis [176,177]. Hypoxic malignant cells are more prone to increase their genetic instability [178], while decreasing the immune response. Moreover, insufficient oxygen supply influences ECM remodeling and stiffness [179], further halting the susceptibility to chemotherapy and radiation therapy [180]. Notably, the enhanced angiogenesis is deemed to counteract the neoplastic metabolic and energetic need, but also shapes the tumor microenvironment and boosts the malignant cells faculty to gain immunosuppression, fueling the cancer progression [181].

The association of cell signaling driving cellular adaptation to hypoxia prompted the investigation on targets that might halt the proliferation of hypoxic tumors if halted. The three pivotal oxygen-dependent molecular mechanism during metabolic adaptation rely on hypoxia inducible factors (HIF members), unfolded protein response (UPR) [182], and mammalian target of rapamycin (mTOR) [8]. Specific targeting of hypoxia in cancer therapy has been extensively investigated and trials exploiting hypoxia-dependent druggable signaling are ongoing [181,183].

Nonetheless, normalizing the tumor vasculature with the judicious use of antiangiogenics can revert this process, directing intervening in oxygen delivery [184,185,186].

As proangiogenic factors typically predominate, tumour perfusion and oxygenation are usually impaired; the re-establishment of physiologic equilibrium aims to vasogenic edema and interstitial pressure reduction, while enhancing the drug delivery and indirectly reducing neoplastic cells shedding and invasiveness [160,187]. A paradigmatic example of in vivo modelling of judicious use of anti-angiogenic treatment has been pioneered by Winkler et al. using anti-VEGFR2 targeting in glioblastoma multiforme (GBM), able to increase pericyte coverage in mature vessels [188], and further corroborated in other tumour types [153,189]. Because of this improvement in the vessels’ structure, functional consequences such as radiation and anti-angiogenic synergism occur during the vessel “normalization window” [188]. The pericyte recruitment parallels angiopoietin-1 (Ang1) and angiopoietin-2 (Ang2) crosstalk. Ang1 promotes vessel maturation and survival through Tie-2 receptor phosphorylation and via the PI3K-AKT-mediated signaling pathway. The development that follows after the formation of immature vessels is mainly due to Ang1 and ephrin B2. Conversely, Ang2 is a context-dependent molecule that counterbalances Ang1 [190,191]. Thus, the Ang1/Ang2 ratio might correlate with vascular normalization [188]. Notably, Ang2 overexpression decreases the prognostic advantage gained by anti-VEGFR strategies [192], uncovering Ang2 to be a rate-determining step for anti-VEGFR treatment. In fact, the dual anti-Ang2/VEGFRs therapy has been shown to enhance the length of the window of vessel normalization in vivo, thus achieving survival improvement and tumour burden reduction upon dual VEGFR2-Ang2 inhibition [193]. These treatment effects of simultaneous VEGFR and Ang2 halting were observed in preclinical models, not in patients and, therefore, must be translated with carefulness. Nonetheless, reprogramming of tumour milieu for immunotherapeutical purposes seems to be conceivable because of the plethora of pathophysiological effects played by VEGF on the immune innate and adaptive compartment [160,194], by enhancing the recruitment and proliferation of cancer tolerogenic Tregs cells [195] and tumour associate macrophages (TAM). Both actors nurse the milieu, making it tolerogenic, and feed into auto-paracrine myeloid-derived suppressor cells (MDSCs) [196] via VEGF and break cytotoxic T lymphocytes’ (CTLs) effector functions [58,197]. Collectively, the abnormal cancer vasculature contributes to immunosuppression in the niche [194,198,199,200] and enhances the shedding of systemic factors hijacking the anti-cancer response [22,198,201].

Current advances in tumour immunotherapy consent to proficiently unleash immune effector cells [202]. What ensues is an immune-supportive skewing, also originating from the vascular normalization [23,203]. Typically, the highest anti-angiogenic doses have been employed at the maximum tolerated doses until PD. Nonetheless, the dosage is key because increasing the amount and a sustained extent of anti-angiogenic therapy are themselves associated with cancer hypoxia and, eventually, PD [23,198,204]. The insights regarding the window of normalized perfusion from vascular normalization depend on the dose and potency of the antiangiogenic therapy. Precisely, the degree of neo-vessels normalization in localized and disseminated cancers is liable determined by the dose of anti-angiogenic compounds and the amount of the angiogenic stimulus in the given neoplasia [204]. Disproportionate perfusion reduction can boost oxidative stress and dissemination potential, while halting the immune infiltrate [205,206]. Therefore, as the stage of normalized cancer oxygen delivery after tailored anti-angiogenic treatment is transitory, the choice of the proper timing matching the vascular normalization “window”, the tailored dose of anti-angiogenic treatment, as well as the most effective immune-modulatory agent appear critical. An elevated concentration and therapy extent of anti-VEGF therapy are associated with decreased cancer oxygen supply and elevated hypoxia [23]. Notably, pre-clinical models uncovered a lower concentration of anti-angiogenic agents to be correlated with sustained vascular normalization [22,198], as low as one-quarter of the conventional dose. Clinical studies corroborated these findings, demonstrating that a decreased dose of anti-VEGF (<3.6 mg/kg, weekly) combined with cytoreduction resulted in improved survival over a high dose (5 mg/kg, week) in subjects suffering from glioblastoma [207,208]. Many attempts have been proposed to unbridle an effective immune response while breaking the vicious cycle between abnormal angiogenesis and immune patrolling actors in aggressive and refractory malignancies [209,210,211] Collectively, the combination of angiogenesis and immunity targeting has been studied a lot in pre-clinical as well as clinical settings, some of them showing promising results [160,212,213]. Overall, the knowledge on the abnormal vasculature and microenvironment provides the backbone for normalization of tumour vasculature strategy, with the judicious use of antiangiogenics and niche reprogramming with the goal of immunotherapy improvement [161].

## 7. Conclusions

Critical mechanisms fostering blood and lymphatic vessels’ formation and facilitating immunosuppression throughout tumor growth and progression have been uncovered. Cancer cells grow and progress through a persistent crosstalk with the neighboring milieu. Next generation techniques sketch at a high resolution such that the new vessels’ formation and immune paresis regularly occur to fuel this vicious cycle. Consequently, state-of-the-art therapeutic strategies merging anti-angiogenic and immune-directed treatments appear to hold promise to shape the neoplastic ecosystem and boost the therapeutic efficacy.

## Figures and Tables

**Table 1:** Endothelial cells function as a gatekeeper for immunological patrolling in solid and hematological malignancies: synthetic overview of the molecular actors. PD-L1, programmed death-ligand 1; IFNγ, interferon gamma; FGF-2, fibroblast growth factor-2; ICAM, intercellular adhesion molecule; VCAM, vascular cell-adhesion molecule; JAM, junctional adhesion molecule; NEU1, epidermal growth factor like domain 7; VEGF, vascular endothelial growth factor; HLA-E, human leukocyte antigen E; ENO-1, Enolase 1; CCL/CXCL, chemokine ligand; TNF, tumour necrosis factor; NO, nitric oxide; TIM3, T-cell immunoglobulin and mucin domain 3; IDO1, indoleamine 2,3-dioxygenase 1; LFA1, lymphocyte function-associated antigen 1; VLA4, very late antigen 4; VE-cadherin, vascular endothelial cadherin; PECAM1, platelet/endothelial-cell adhesion molecule 1; ESAM, endothelial cell-selective adhesion molecule.

Proangiogenic Molecules *	Soluble Factors *	Immune Checkpoints	Major Histocompatibility Complex (MHC)	Adhesion Molecules *
***FGF2*** Modulate selective up- and down-regulation [56,103,104] of adhesion molecules (ICAM [30,105], VCAM [106], JAMs [35,107,108])	***Chemokines (CCL2/18, CXCL10/11, CXCL4)*** Deregulated chemokines, halting immune effector surveillance and attracting immune tolerogenic cells [27,43,105,109,110]	***PD-L1/2*** Cancer endothelium, also express immune checkpoints: a cross talk between aberrant vasculature, immune, and cancer cells creates an immune permissive tumor milieu [58,101,111,112,113,114]	***MHC I*** Often overexpressed within the tumor niche, where the cancer associated endothelium is characterized by a lack of co-stimulatory molecules (B7.1–and B7.2) [58,115,116,117]	***Selectin-mediated leukocyte rolling*** **E-selectin/P-selectin** Orchestrate leukocyte recruitment. [79,118,119]
***NEU1*** Induces a decreased adhesion molecule expression and boosts angiogenesis via NOTCH pathway [120,121,122]	***Cytokines (IFNγ,TNFα)*** unresponsiveness and anergy along with PD-L1 overexpression [112,123,124,125,126,127]	***ENO-1*** acts both as a glycolytic enzyme and a plasminogen receptor expressed on the cell surface of tumor cells. Surface ENO1 plays a crucial role in cancer metabolism, tumor invasion and immune suppression in the cancer immune-microenvironment [35,128]	***MHC II*** Can be decreased on tumor infiltrating vessels, thus contributing to an immune tolerogenic niche [117]	***Integrin-mediated leukocyte rolling:*** ***ICAM1 binds to LFA1 (αLβ2 integrin);*** ***VCAM1 binds to VLA4 (α4β1 integrin)*** Multiple functions [27] ** Function
***VEGF-A/C*** Vascular endothelial growth factors induce cell phenotype changes, recruiting immune suppressive cells [58,62,129,130]	***NO*** Directly and indirectly affect effective immune response by altering leukocyte infiltration and suppressing CD8+ T cells [131,132,133,134]	***IDO1 and TIM3*** Immune regulatory checkpoints overexpress in cancer endothelial cells upon cytokines stimulation (i.e., *IFNγ*) able to induce T cells programmed cell death and cell cycle arrest, respectively [114]	***HLA-E*** CD8+T cells infiltration in ovarian cancer correlated with improved better survival when HLA-E expression is decreased [135]	***VE-cadherin and intracellular membrane compartments, containing PECAM1, JAMs, ESAM, ICAM2, and CD99*** promote paracellular migration [27,136,137,138] ^#^

* Molecules with demonstrated immunological function influencing microenvironment patrolling are summarized. ** Endothelial cells express selectins and integrins, the most important leukocyte adhesion cascade tumour-associated endothelial cells, express lower levels of cell adhesion molecules, promoting endothelial anergy and reducing the ability of effector T cells to infiltrate tumours. ^#^ Abherrant expression of cancer associated vessels surface proteins contribute to the hypoxia and acidosis, which, in turn, enhance adhesion molecules’ expression, recruiting immune-suppressive cells and conversely excluding effector T cells, by downregulating key integrin and selectins. Adhesion molecules are pivotal in gatekeeping function of endothelial-mediated transmigration.

## Abbreviations

CCL	Chemokine ligand
CXCL	Chemokine ligand
ENO1	Enolase1
ESAM	Endothelial cell-selective adhesion molecule
FGF2	Fibroblast growth factor 2
ICAM	Intercellular adhesion molecule
IDO1	Indoleamine 2,3-Dioxygenase 1
HLA-E	Human leukocyte antigen E
IFNγ	Interferon gamma
JAMs	Junctional adhesion molecules
LFA1	Lymphocyte function-associated antigen 1 (also known as αLβ2-integrin)
MHC	Major histocompatibility complex
NEU1	Epidermal growth factor like domain 7 (Egfl7)
NO	Nitric oxide
PD-L1/2	Programmed death-ligand 1/2
PECAM1	Platelet/endothelial-cell adhesion molecule 1
TIM3	T-cell immunoglobulin and mucin domain 3
TNFα	Tumour necrosis factor alpha
VCAM	Vascular cell-adhesion molecule
VE-cadherin	Vascular endothelial cadherin
VEGF	Vascular endothelial growth factor
VLA4	Very late antigen 4 (also known as α4β1-integrin)
